# The effect of topical olive oil on the healing of foot ulcer in patients with type 2 diabetes: a double-blind randomized clinical trial study in Iran

**DOI:** 10.1186/s40200-015-0167-9

**Published:** 2015-04-29

**Authors:** Morteza Nasiri, Sadigheh Fayazi, Simin Jahani, Leila Yazdanpanah, Mohammad Hossein Haghighizadeh

**Affiliations:** Department of Nursing and Midwifery, Diabetes Research Center, Ahvaz Jundishapur University of Medical Sciences, Ahvaz, Iran; Health Research Institute, Diabetes Research Center, Ahvaz Jundishapur University of Medical Sciences, Ahvaz, Iran; Department of Health, Ahvaz Jundishapur University of Medical Sciences, Ahvaz, Iran

**Keywords:** Diabetic foot ulcer, Ulcer healing, Olive oil, Herbal products

## Abstract

**Background:**

Diabetic Foot Ulcer (DFU) is the most costly and devastating complication of diabetes mellitus which can lead to infection, gangrene, amputation, and even death if the necessary care is not provided. Nowadays, some herbal products have shown therapeutic effects on healing of DFU. So, this study aimed to assess the effects of topical olive oil on the healing of DFU.

**Methods:**

This double-blind randomized clinical trial study was conducted in Diabetes Clinic of Ahvaz Golestan hospital, Iran, in 2014. Thirty-four patients with DFU of Wagner’s ulcer grade 1 or 2 were enrolled in this study. Patients who were randomly assigned to intervention group (n = 17) received topical olive oil in addition to routine cares, whereas patients in control group (n = 17) just received routine cares. Intervention was done once a day for 4 weeks in both groups, and in the end of each week; the ulcers were assessed and scored. Data was collected by demographic and clinical characteristics checklists as well as diabetic foot ulcer healing checklist, and was analyzed by SPSS version 19 software using descriptive (mean and standard deviation) and analytic (student’s sample t-test, chi-square and repeated-measures analysis of variance) statistics.

**Results:**

At the end of 4^th^ week, there was a significant differences between two groups regarding to 3 parameters of ulcer including degree (P = 0.03), color (P = 0.04) and surrounding tissues (P < 0.001) as well as total status of ulcer (P = 0.001), while related to ulcer drainages no significant difference was seen between the two groups (P = 0.072). At the end of the follow up, olive oil significantly decreased ulcer area (P = 0.01) and depth (P = 0.02) compared with control group. Complete ulcer healing in the intervention group was significantly greater than control group (73.3% vs. 13.3%, P = 0.003) at the end of follow up. Also, there were no adverse effects to report during the study in intervention group.

**Conclusions:**

Our results indicated that olive oil in combination with routine cares is more effective than routine cares alone, and is without any side effect. However, further studies are required in the future to confirm these results.

**Trial registration:**

IRCT2014083014251N2.

**Electronic supplementary material:**

The online version of this article (doi:10.1186/s40200-015-0167-9) contains supplementary material, which is available to authorized users.

## Background

Diabetic Foot Ulcer (DFU) is one of the most common and devastating complications of Diabetes Mellitus (DM), which has indicated an increasing trend in the past decades [1]. Recent investigations have shown that more than 15% of patients with DM had DFU during their lifetime [2]. Although the accurate figures are difficult to obtain for the prevalence of DFU, its prevalence has reported 4% to 27% in different parts of the world [3-6].

DFU mainly caused by ischemic, neuropathic or combined neuroischemic abnormalities [7]. The healing of ulcer in patients with diabetes is reported to be poor [8]. It is reported that a considerable number of patients with DFU remain unhealed after 12 weeks of treatment, and in treatable cases healing occurs in 2 or 5 months virtually [9]. Based on one study conducted in the USA, standard care heals only between 24% and 31% of DFU [10]. Ultimately, unhealed DFU in the most cases lead to infection, gangrene, amputation, and even death if the necessary care is not provided [11]. The risk of lower extremity amputation reports 15 to 46 times higher in patients with diabetes than in persons without diabetes [2]. Furthermore, non-healing ulcers affect patient quality of life and productivity and represent a substantial financial burden on the health care system [12,13]. So, healing of ulcer in patients with diabetes can prevent the most serious complications of this problem.

The primary management goals for DFU are to obtain wound closure as expeditiously as possible [14]. As diabetes is a multi-organ systemic disease, all comorbidities that affect wound healing must be managed by a multidisciplinary team for optimal outcomes with DFU. Based on National Institute for Health and Clinical Excellence (NICE) strategies, the management of DFU should be done immediately with a multidisciplinary team that consists of a general practitioner, a nurse, an educator, an orthotic specialist, a podiatrist, and consultations with other specialists such as vascular surgeons, infectious disease specialists, dermatologists, endocrinologists, dieticians, and orthopedic specialists. Multi-disciplinary approaches for management of DFU include control of patient’s blood glucose, antibiotics therapy, off-loading devices, wound debridement, advanced dressings as well as surgery in selected cases [15,16]. Furthermore, many adjunctive therapies could be used for rapid healing of DFU including hyperbaric oxygen therapy (HBOT) [17], Negative Pressure Wound Therapy (NPWT) [18], recombinant Human Platelet-Derived Growth Factor-BB (rPDGF-BB) [19], Electrical Stimulation (ES) [20] and Bio-Engineered Skin (BES) [21]. In spite of these various options for healing of DFU, studies showed the poor healing of DFU [9,10]. Besides, the rising costs of these approaches impose a lot of costs to patients and health care systems, and urge the need of new therapeutic agents [12]. Therefore, design of low cost and effective methods to heal DFU seems to be essential.

To date, various herbal products have been used in the management and treatment of DFU. Some studies have shown that herbal products provide accelerated healing and decrease the rate of recurrent ulcer complications in patients with diabetes [22-24]. Today, therapeutic effects of some herbal products such as Semelil (ANGIPARS™) [22,25], Radix Astragali (RA) and Radix Rehmanniae (RR) [26], Aloe vera [27] as well as Tangzu Yuyang ointment [28] are discussed in researches for treating of DFU. One of other herbal extracts that its usefulness as external administration in healing of DFU has been reported in previous studies is olive oil [29-32]. In two case report studies in Iran, the administration of Propolis and olive oil extract and application of honey and olive oil mixture reported to be effective in healing of DFU [29,30]. Also, in two quasi-experimental studies that were conducted at Egypt, results showed the effectiveness of ozonated olive oil ointment dressing technique on the healing of DFU [31,32]. However, in one of these studies, no significant difference was seen in healing of ulcer between the studied groups after the end of the follow up [32]. Therefore, due to conflicting results of the researches that have assessed the effects of olive oil on the healing of DFU and so that most founded studies in this regards are case report and quasi-experimental in design, and also regarding to the important role of DFU management, the present study aimed to assess the effect of topical olive oil on the healing of DFU as a clinical trial study to find a novel approach in treatment of this kind of ulcer.

## Methods

### Study design

This double-blind randomized clinical trial study was conducted on patients with DFU who referred to Diabetes Clinic of Ahvaz Golestan Hospital, Iran, from March 1^St^ through September 30^th^, 2014. All patients with type 2 diabetes who presented with one ulcer were entered into this study. Inclusion criteria included: age of 30-65 years, Body Mass Index (BMI) of 18 to 35, having foot ulcer with grade 1 and 2 of Wagener’s classification on toes, soles, heels or dorsum of the feet (not over the pressure points of the feet), which remained open without healing and had not shown improvement for more than 3 months, lack of any history of addiction and cigarette and alcohol abuse as well as lack of participation in any diabetes education and foot preventive education, not receiving drugs which may interact with the study therapeutic protocol (through delaying the healing process) such as glucocorticoids, immunosuppressive and cytotoxic drugs and free from any chronic diseases other than diabetes that may affect the healing of ulcers such as cancers, congestive heart failure, end stage renal disease and liver failure. Patients with foot gangrene that needed amputation as well as osteomyelitis that needed antibiotic therapy during the study were excluded from the study. Also patients who preferred to receive the treatment out of the study, inappropriate follow up by the patients (missing follow-up more than two times), and the patient’s desire to withdraw in each phase of the study were considered as others exclusion criteria.

### Sample size

The sample size of this study was calculated by the same previous study [30], which indicated that 90% of patients in the study group had partial ulcer healing after 4 weeks of follow up as compared to 35% in the control group. Based on the results of this study and using the below formula with confidence level of 95%, the number of needed samples was calculated as 13.67 patients that for getting more confident results we considered 17 subjects in each group.

$$ \operatorname{n}=\frac{{\left({z}_{1-\frac{a}{2}}+{z}_{1-\beta}\right)}^2\left[{p}_1\left(1-{p}_1\right)+{p}_2\left(1-{p}_2\right)\right]}{{\left({p}_1-{p}_2\right)}^2}=\frac{{\left(1.96+1.65\right)}^2\left[0.35(0.65)+0.9(0.1)\right]}{{\left(0.35-0.9\right)}^2}=13.67\tilde{=}14 $$

### Data collection

Data was collected with two researcher-made checklists as well as Wagner system of ulcer classification. The first checklist was related to demographical and clinical characteristics of patients including: age, sex, marital status, educational level, occupation, kind of prescribed medications, BMI, duration of diabetes, Fasting Blood Sugar (FBS) and glycosylated hemoglobin (HbA1C). In this study, BMI was calculated by measuring patients’ height and body weight and then use the following equation [BMI = weight / (height^2^) = Kgm/m^2^]. In order to measure FBS and HBA1C, a 5 ml blood sample was obtained from all subjects after an overnight fasting and the blood samples were investigated for these variables by FBS test and High Performance Liquid Chromatography (HPLC) method, respectively.

The second tool was diabetic foot ulcer healing checklist that consisted site, grade, size and duration of ulcer, vascular and neuropathy status of ulcer, weekly ulcer healing assessment scale, status of ulcer healing and adverse effects at the end of the study (Additional file 1). Ulcer size was considered as ulcer surface area and depth. Ulcer surface area was measured with computerized planimetry (Sigma Scan Pro; SPSS Inc, Chicago, Ill), and ulcer depth was measured at the deepest part of the ulcer with a sterile probe. For estimating ulcer size photographs (Macro 3 SLR Camera; Polaroid Corp, Waltham, Mass) were taken with standardized lighting and positioning for each patient. The diagnosis of lower-extremity vascular insufficiency was made clinically on the basis of absence of both pedal pulses of the involved foot and/or an ankle-brachial pressure index of <0.9. Neuropathy was considered as vibration perception more than 25 v by neurothesiometer and/or loss of pressure sensation in more than one point in 10 g monofilament examination. Ulcer healing assessment scale evaluated 4 ulcer’s parameters including degree, color, surrounding tissues and drainages as well as total ulcer status weekly. Based on this scale, each parameter obtained 100 scores, and the total ulcer status scores range from 50 to 400. Based on this scale, the higher the score the better healing (Additional file 2). The status of ulcer healing at the end of the 4^th^ week was considered as complete, partial, lack of healing and deterioration according to the scores that was obtained by ulcer healing assessment scale at the end of follow up. Complete ulcer healing was considered as cases that their ulcers had 400 scores, and partial ulcer healing was considered as increase of ulcers scores at least 30 scores more than the first week. Lack of healing was considered as lack of any changes in ulcers scores and deterioration of ulcer was considered as decrease of ulcers scores at least 10 scores less than the first week. The incidence of adverse events was evaluated by recording all observed or volunteered adverse events. For this purpose, study related adverse events inducing ulcer area sensitivity, bleeding, infection or pain during treatment were monitored by daily evaluation. For patients who withdrew or patients lost to follow-up, adverse events were acquired by telephone. In order to determine the scientific validity of the researcher-made checklists, content validity was used. For this purpose, after studying books and other resources related to the subject, a checklist was prepared and then presented to 10 faculty members of medical surgical nursing and medicine (endocrinologist) of Ahvaz Jundishapur University of Medical Sciences, Iran, and after collecting their suggestions, final checklist was prepared.

In this study, Wagner system was used for ulcer classification that its reliability and validity was proven by previous study [33]. Based on this system, ulcer classify into 5 grades. Grade 1 ulcers are superficial ulcers involving the full skin thickness, but no underlying tissues. Grade 2 ulcers are deeper, penetrating down to ligaments and muscle, but not involving bone or abscess formation. Grade 3 ulcers are deep ulcers with cellulitis or abscess formation, often complicated with osteomyelitis. Ulcers with localized gangrene are classified as Grade 4, and those with extensive gangrene involving the entire foot are classified as Grade 5 [34].

### Interventions

This study was approved by ethics committee of Ahvaz Jundishapur University of Medical Sciences, Iran, with code No. ajums.REC.1393,61, and was registered in Iranian Registry of Clinical Trials (IRCT) with code No. IRCT2014083014251N2. After obtaining an informed consent from all of the patients and providing verbal explanation about the research and assurance of confidentiality and anonymity, 34 patients were enrolled based on inclusion criteria and were randomly divided into two groups of control and intervention according to the day of admission. Seventeen patients who admitted in odd days were assigned to the intervention group and seventeen patients who admitted in even days were assigned to control group. Matches of the patients in both groups were done related to age, sex, ulcer degree and site as well as BMI and FBS.

At initial meeting that was considered as baseline (week 0), assessment of patients’ ulcer was done for both groups by the same trained physician. Then, due to ulcer healing could affected by status of nutrition as well as immune defense’s system, all patients were educated on diet, prescribed medications use and management of the ulcer in one section during 2 hours. After that, patients in intervention group received topical olive oil in addition to routine cares, whereas patients in control group just received routine cares.

In present study, routine cares was implemented based on cares protocol for DFU patients that was developed by the physicians and nurses of the recruitment center. According to this protocol, the periphery and the center of the ulcers was irrigated and cleaned with 1000 ml sterile normal saline solution (0.9%) every day and after making dry was dressed with sterile gauze and latex-free tape. Also based on this protocol, all of the patients received oral antibiotics and local debridement as well as off-loading with shoes modified whenever needed. Furthermore, FBS and HbA1c of patients was tested weekly and before and after intervention, respectively.

In this study, refined olive oil prepared by Loyeh Product and Services, Tehran, Iran, was used. At first this oil was poured to surface of patients’ ulcer using a syringe based on the area and depth of the ulcer (about 1 mm thick), and then to ensure ulcer contact with the oil, a gauze soaked with oil was applied to ulcer and then was covered with latex-free tape. External administration of this kind of olive oil hasn’t shown any side effects on DFU healing in previous study [25,26]. Also, based on our pilot study that was conducted on 5 patients (not included in main sample) the topical usage of this oil showed no adverse effects.

The intervention was done every day until 4 weeks in both groups by the same trained and experienced researcher in the recruitment center. At the end of each week, the appearance of ulcer was assessed and scored again with ulcer healing assessment scale, and at the end of 4^th^ week; photograph was taken and measurement of ulcer size was estimated in both groups by the same trained physician. Both pre- and post-assessment and scoring was done by the same trained physician who was not aware of the groups’ assignment. Also, the statistic analyzer who analyzed the data was unaware of groups’ assignment. So, this study was blinded in this regard.

### Statistical analysis

For doing statistical analysis, the Statistical Package for the Social Sciences (SPSS) version 19 (SPSS Inc, Chicago, Illinois) software was used. Descriptive statistical tests (mean, standard deviation, frequency and percentage) were used for demographic and clinical characteristics. Independent sample t-test was used to compare quantitative variables in the both groups. Also, chi-square test was performed for qualitative variables. Repeated-measures analysis of variance was used to test for significance of changes in ulcer size between the two groups. P < 0.05 was considered to indicate statistical significance.

## Results

### Demographic and baseline characteristics

**Figure 1:**
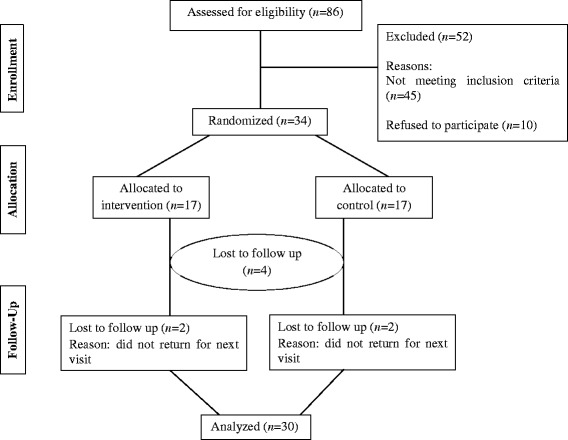
Flow of participants through the trial.

Of the 34 patients enrolled in this study, 30 adhered to the protocol. Two patients in control group and two patients in intervention group were excluded from the study due to loss of follow up. Therefore, 30 patients (15 patients in the intervention group, and 15 patients in control group) were included in the final analysis. The flow chart of the patients’ recruitment is shown in Figure 1. In this study just one patient in control and one patient in intervention group received off-loading with shoes modified.

**Figure 2:**
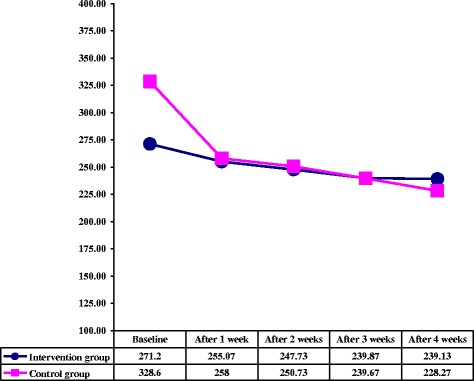
Comparison of two groups according to fasting blood sugar at baseline and during follow up visits.

The patients’ age in both studied groups was ranged from 40-65 years with a mean age of 53.8 ± 1.3 and 52.6 ± 9.13 years for intervention and control groups, respectively. Duration of diabetes (years) and ulcer (months) were 12.73 ± 7.48 and 50.0 ± 28.65 in intervention groups and were 14.93 ± 10.38 and 44.93 ± 30.37 in control group, respectively. Based on student’s sample t-test, there was no significant difference regarding the patients’ age (P = 0.877) and their duration of diabetes (P = 0.511) and ulcer (P = 0.642) between the groups. Amongst patients, 4 patients (26.66%) in intervention and control groups had ischemic and the remaining 11 patients (73.34%) had neuropathic ulcers that in this regard no significant difference was seen between two groups. Other demographical and clinical characteristics of the patients are shown in Table 1, which indicate no statistically significant differences between the two groups. The mean of HbA1C at initial visit was 9.97 ± 3.08 and 9.58 ± 2.78, and at the end of follow up was 9.08 ± 2.78 and 9.05 ± 2.84 in intervention and control groups, respectively. There was no statistical significant difference between two groups at initial visit (P = 0.726) and at the end of follow up (P = 0.721) regarding the HbA1C. The Figure 2 indicates that no significant difference between the two groups was existed regarding to FBS at the beginning of the study and at the beginning of the 4 weeks of followed up (P > 0.5).

**Table 1:** **Demographic and clinical characteristics of patients in both groups**

**Group**		**Intervention (n = 15)**		**Control (n = 15)**		**P–Value**
**Demographic characteristic**		**Number**	**Percent (%)**	**Number**	**Percent (%)**	
**Sex**	**Female**	10	66.7	9	60.0	0.705
	**Male**	5	33.3	6	40.0	
**Educational level**	**Illiterate**	11	73.4	8	53.4	0.445
	**Less than a diploma**	2	13.3	3	20.0	
	**Diploma**	2	13.3	2	13.3	
	**Collegiate**	0	0.0	2	13.3	
**Marital status**	**Married**	15	100.0	14	93.3	0.309
	**Divorced**	0	0.0	1	6.7	
**Occupation**	**Housewife**	9	60.0	8	53.4	0.942
	**Working (unskilled, skilled and clerical work)**	3	20.0	3	20.0	
	**Not working**	1	6.7	2	13.3	
	**Retirement**	2	13.3	2	13.3	
**Medications**	**Insulin**	6	40.0	10	66.7	0.065
	**Hypoglycemic agents**	11	73.3	6	40.0	0.143
**BMI**	**18-24**	2	13.3	3	20.0	0.755
	**25-29**	4	26.7	5	33.3	
	**30-35**	9	60.0	7	46.7	
**Site of ulcer**	**Sole**	3	20.0	4	26.7	0.946
	**Toe**	10	66.7	9	60.0	
	**Dorsum of the feet**	2	13.3	2	13.3	
**Grade of ulcer**	**Wagner 1**	6	40.0	7	46.7	0.946
	**Wagner 2**	9	60.0	8	53.3

### Ulcer size

Baseline ulcer surface area (cm^2^) was 1.17 ± 0.69 and 0.87 ± 0.26 in control and intervention groups respectively. Baseline ulcer depth (cm) was 0.34 ± 0.07 and 0.24 ± 0.05 in control and intervention groups respectively. Initial surface area (P = 0.66) and depth (P = 0.28) were not statistically different between the two groups.

At the end of the follow up, olive oil significantly decreased ulcer area (P = 0.01) and depth (P = 0.02) compared with control group. At the end of the study period, the ulcer area had changed by −54.7 ± 28.8% in the olive oil group (P = 0.02 vs at the start of the study) and by +2.7 ± 47.2% in the control group (P = 0.18). The ulcer depth had changed by −60.1 ± 13.8% in the olive oil group (P = 0.004 vs at the start of the study) and −29.6 ± 12.6% in the control group (P = 0.04).

### Ulcer parameters and total ulcer status scores

The data in Table 2 shows ulcer parameters and total ulcer status scores in both studied groups at baseline and during follow up period. Based on this table, results indicated no significant difference between the two groups regarding to all ulcer parameters and total ulcer status at baseline, while after 1^st^ week of follow up a significant difference was seen regarding to ulcer color and surrounding tissues. Furthermore, a significant difference was seen regarding to ulcer degree after 2^nd^ week of follow up between the two groups. However, regarding to ulcer drainages results showed no significant difference between studied groups in none of the 4 weeks of follow up. Total ulcer status scores showed significant difference between the two groups at the end of the 1^st^, 2^nd^ and 4^th^ weeks of follow, while at the end of 3^rd^ week no significant difference was observed in this regard between two groups.

**Table 2:** **Comparison of mean and SD of ulcer parameters and total ulcer status scores at the baseline and during follow up visits in each group**

**Ulcer parameters**	**Time**	**Intervention (n = 15)**		**Control (n = 15)**		**P–Value**
		**Mean**	**SD**	**Mean**	**SD**	
Degree	Baseline	69.0	11.83	61.0	17.54	0.154
	After 1 week	79.33	10.15	69.33	17.30	0.064
	After 2 weeks	87.33	9.79	74.33	17.20	0.017
	After 3 weeks	92.33	9.79	80.0	16.47	0.019
	After 4 weeks	96.66	6.17	82.66	15.56	0.03
Color	Baseline	66.0	9.10	65.33	12.45	0.868
	After 1 week	84.0	9.85	69.0	11.68	0.001
	After 2 weeks	90.0	10.1	78.66	14.57	0.019
	After 3 weeks	94.66	6.39	86.0	11.83	0.019
	After 4 weeks	97.33	4.57	86.66	12.34	0.04
Surrounding tissues	Baseline	67.0	15.32	69.0	11.68	0.691
	After 1 week	81.33	12.31	73.33	8.16	0.045
	After 2 weeks	90.33	9.72	79.33	12.22	0.011
	After 3 weeks	94.66	6.11	83.00	13.33	0.005
	After 4 weeks	97.33	4.57	83.0	13.33	< 0.001
Drainages	Baseline	86.0	14.54	84.0	16.81	0.730
	After 1 week	93.33	9.75	87.33	15.33	0.212
	After 2 weeks	97.33	7.03	92.66	13.34	0.241
	After 3 weeks	98.86	5.16	94.00	10.55	0.135
	After 4 weeks	100	00.00	96.00	8.28	0.072
Total ulcer status	Baseline	288.00	40.52	277.33	35.55	0.450
	After 1 week	342.00	33.63	301.67	35.89	0.004
	After 2 weeks	365.00	29.82	325.00	43.91	0.007
	After 3 weeks	373.67	37.48	343.00	26.20	0.056
	After 4 weeks	391.33	15.05	348.00	43.08	0.001

### Ulcer healing status

**Figure 3:**
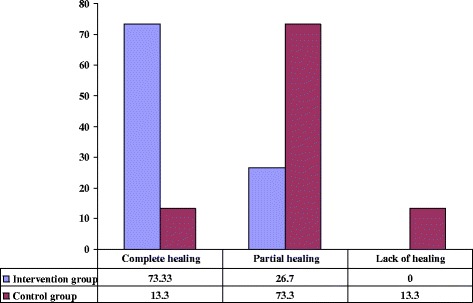
Comparison of two studied groups according to ulcer healing status after 4 weeks (the end of follow up).

**Figure 4:**
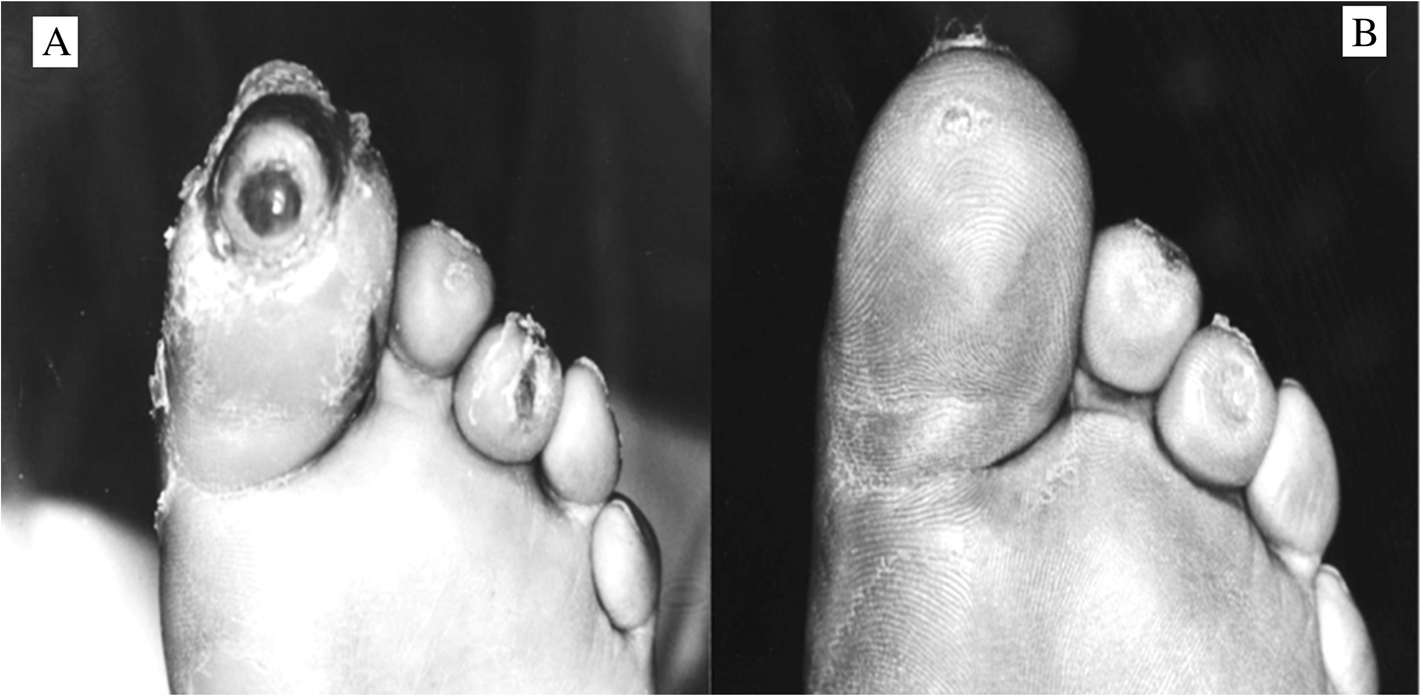
Complete healing in a patient treated with topical olive oil before intervention **(A)** and at the end of follow up **(B).**

Figure 3 shows comparison between the two studied groups according to ulcer healing status after 4^th^ week (the end of follow up). Majority of patients in the intervention groups (73.3%) had complete ulcer healing, while 26.7% of them had partial healing. In control group, it was found that at the end of follow up period, 13.3% of patients had complete ulcer healing and 73.3% had partial ulcer healing, while 13.3% of them complained of lack of healing. A statistical significant difference was found between the intervention and control groups in relation to healing process after 4^th^ week (P = 0.003). Figure 4 shows one of the patients in intervention group who had complete ulcer healing after 4 weeks of intervention.

### Adverse effects

All patients tolerated treatment by topical olive oil very well and there were no adverse effects to report during the study.

## Discussion

DFU is a common, expensive and debilitating problem among diabetic patients that may lead to infection and amputation [11]. Therefore, today several different methods have been studied to achieve better results in the treatment of this sort of ulcers [14]. Recently, studies have shown therapeutic effects of some herbal products on healing of ulcer in patients with DFU [22-28]. In consistent with previous studies [29-32], findings of this research showed that olive oil as an herbal product could be effective in healing of DFU. Based on our results, however total ulcer status showed no significant difference between the two groups at the end of the 3^rd^ week, in the 1^st^, 2^nd^ and 4^th^ weeks of follow up significant difference was observed in this regard between the two groups, which it difference was in favor of intervention group in increase of mean scores. So, it shows the potential efficacy of topical olive oil as an adjunct to routine wound care on healing of DFU. In this regard, our result is to some extent similar to Elshenawie *et al,* study which assessed the effects of ozonated olive oil ointment dressing technique on the healing of superficial and deep DFU at Egypt. Based on the results of their study, at the end of the 2^nd^, 3^rd^ and 4^th^ weeks of follow up period the ozonated olive oil dressing had significant effect on ulcer healing than conventional dressing technique (P < 0.05) [31], which is in line with our results except in the 3^rd^ week. In another similar study that was performed by Aziza *et al,* results indicated that only at the end of the 2^nd^ week of follow up period the ozonated olive oil dressing had significant effect on ulcer healing than conventional dressing technique (P < 0.05) [32], which is compatible with our result in the 2^nd^ week, while is not in line with our result in the 1^st^ and 4^th^ weeks of follow up.

In present study, 73.3% of the patients in the intervention group had complete ulcer healing as compared to 13.3% in the control group at the end of the follow up, which reveals that patients who treated with olive oil had better healing process. In this regard, our result is to some extent consistent with the results of to Elshenawie *et al,* which indicated that 60% of patients in the study group had complete ulcer healing at the end of follow up period as compared to 0% in the control group [31]. However, the results of Aziza *et al,* showed that 88.0% of patients in the study group had complete ulcer healing as compared to 80.0 % in the control group at the end of follow up period [32], which is not in accordance with our result in control group. Regarding to partial ulcer healing, our results showed that 26.7% of the patients in the intervention group had partial ulcer healing as compared to 73.3% in the control group at the end of the follow up, which is in line with the results of to Elshenawie *et al,* that showed most of patients (66.7%) in the control group had partial ulcer healing at the end of follow up [31], but is inconsistent with the results of Aziza *et al,* that showed only 12.0% had partial ulcer healing in the control group [32]. In present study, no patients complained of lacking ulcer healing in the intervention group, while 13.3% of the patients in the control group had unhealed ulcer at the end of follow up period, which is consistent with the results of to Elshenawie *et al,* [31] and Aziza *et al,* [32] that showed 33.3% and 4.0% of patients respectively complained of lacking ulcer healing in control groups at the end of follow up. The discrepancies between our results and above mentioned studies may be due to administration of different type of olive oil and using different questionnaires. Beside, in our study so that care techniques are different among health care providers, all intervention was done by trained researcher but in Aziza *et al,* [32] study the intervention was done by patients. So, it’s may be another reason for differences between our results.

Regarding to ulcer size, olive oil significantly decreased ulcer area and depth compared with control group at the end of the follow up. Unfortunately, we couldn’t find similar study to compare our results.

Despite the positive clinical evidence, the pathophysiological basis of live oil benefits on ulcer healing is not yet clear. Investigations have revealed that olive oil probably improves total tissue blood flow [35,36] and reduces inflammation [37,38], thus causes ulcer healing. Olive oil is composed of 98% triglycerides, including predominantly monounsaturated oleic acids, which their anti-inflammatory properties have been proven to be essential for skin maintenance, as such properties are similar to ibuprofen (recent studies attribute this to Oleocanthal), and this may accelerate the recovery and healing process of ulcers [39,40]. Furthermore, owing to the high concentration of polyphenols, which are natural antioxidants, included in olive oil, its use mitigates the inflammatory process and increase blood flow, thus helps in ulcer healing [40,41].

A major concern during the treatment with herbal medications is the unpredicted side effects such as allergic reactions. Fortunately, similar to the previous studies [29-32] no significant side effects were observed in patients treated with olive oil which indicates that this oil could well become a part of routine therapy for DFU. So, this oil should be more assess by researcher in educational and research centers due to it is found abundantly in Iran and compared to the chemical drugs, has no adverse effects and is quite cheap.

Some limitations in this study should be noted. Firstly, the patients’ response to ulcer healing is affected by genetic differences that were out of researchers’ control. Secondly, due to the waxy nature of the olive oil we couldn’t use placebo in control group. Thirdly, some co-founding factors such as impact from neuropathy, off-loading and ulcer pressure have not been excluded in this study. Fourthly, follow up time in this study was too short and we couldn’t follow up patients until complete healing. Finally, this study is limited by virtue of a small patients’ population that may create a low power of statistical analysis. So, it’s suggested that future study pay more attention to these limitations and conduct their investigations with other kinds of olive oil and treat and follow up patients until complete healing.

## Conclusion

Our results indicated that olive oil in combination with routine cares is more effective than routine cares alone, and is without any side effect. However, further studies are required in the future to confirm these results.

## Additional files

Additional file 1:
**Attachment 1.** Diabetic foot Ulcer healing assessment scale. Additional file 2:
**Diabetic foot Ulcer healing assessment scal.**

## Abbreviations

DFU: Diabetic Foot Ulcer
DM: Diabetes Mellitus
NICE: National Institute for Health and Clinical Excellence
HBOT: Hyperbaric oxygen therapy
NPWT: Negative Pressure Wound Therapy
rPDGF-BB: recombinant Human Platelet-Derived Growth Factor-BB
ES: Electrical Stimulation
BES: Bio-Engineered Skin
RA: Radix Astragali
RR: Radix Rehmanniae
BMI: Body Mass Index
FBS: Fasting Blood Sugar
HbA1C: Glycosylated hemoglobin
HPLC: High Performance Liquid Chromatography
