# Supplementary material for: The effect of topical olive oil on the healing of foot ulcer in patients with type 2 diabetes: a double-blind randomized clinical trial study in Iran
Source: J Diabetes Metab Disord. 2015 Apr 29;14:38. doi: 10.1186/s40200-015-0167-9 (PMC4428202; doi:10.1186/s40200-015-0167-9)
Supplement: Additional file 1: — Attachment 1. Diabetic foot Ulcer healing assessment scale. [file 40200_2015_167_MOESM1_ESM.docx]

***Attachment 1:* *Diabetic foot Ulcer healing assessment scale***

| **Total score** | **Ulcer Parameters** | **Distribution of scores** | | | | | | | | | | | | | | | | |
| --- | --- | --- | --- | --- | --- | --- | --- | --- | --- | --- | --- | --- | --- | --- | --- | --- | --- | --- |
| **100** | **Degree** | **Stage** | **0** | **1** | **2** | **3** | **4** | **5** | **6** | **7** | **8** | **9** | **10** | **11** | **12** | **13** | **14** | **15** |
|  |  | **Score** | **100** | **90** | **90** | **80** | **75** | **65** | **65** | **55** | **50** | **40** | **40** | **30** | **25** | **15** | **15** | **10** |
| **100** | **Color** | **Center** | **Total healing** | | | **Red** | | | **Yellow** | | | **Necrotic** | | | **Necrotic + Red** | | | |
|  |  |  | **50** | | | **40** | | | **30** | | | **20** | | | **10** | | | |
|  |  | **Surroundings** | **Total healing** | | | **Red** | | | **Yellow** | | | **Necrotic** | | | **Necrotic + Red** | | | |
|  |  |  | **50** | | | **40** | | | **30** | | | **20** | | | **10** | | | |
| **100** | **Surrounding tissues** | **Color** | **Normal** | | | **Red** | | | **Pale** | | | **Cyanotic** | | | **-** | | | |
|  |  |  | **25** | | | **20** | | | **15** | | | **10** | | | **-** | | | |
|  |  | **Hotness** | **Yes** | | | **No** | | | **-** | | | **-** | | | **-** | | | |
|  |  |  | **0** | | | **25** | | | **-** | | | **-** | | | **-** | | | |
|  |  | **Edema** | **Yes** | | | **No** | | | **-** | | | **-** | | | **-** | | | |
|  |  |  | **0** | | | **25** | | | **-** | | | **-** | | | **-** | | | |
|  |  | **Sense** | **No** | | | **Decreased** | | | **Yes** | | | **-** | | | **-** | | | |
|  |  |  | **0** | | | **15** | | | **25** | | | **-** | | | **-** | | | |
| **100** | **Drainages** | **Color** | **Without Drainages** | | | **Serosal** | | | **Bloody** | | | **Yellow** | | | **Green** | | | |
|  |  |  | **40** | | | **30** | | | **30** | | | **20** | | | **10** | | | |
|  |  | **Odor** | **No** | | | **Yes** | | | **-** | | | **-** | | | **-** | | | |
|  |  |  | **20** | | | **0** | | | **-** | | | **-** | | | **-** | | | |
|  |  | **Amount** | **Without Drainages** | | | **Low** | | | **Moderate** | | | **Much** | | | **-** | | | |
|  |  |  | **40** | | | **20** | | | **20** | | | **10** | | | **-** | | | |
| **Total score (total healing) = 400 (more score= more healing)** | | | | | | | | | | | | | | | | | | |
