# Supplementary material for: The effect of topical olive oil on the healing of foot ulcer in patients with type 2 diabetes: a double-blind randomized clinical trial study in Iran
Source: J Diabetes Metab Disord. 2015 Apr 29;14:38. doi: 10.1186/s40200-015-0167-9 (PMC4428202; doi:10.1186/s40200-015-0167-9)
Supplement: Additional file 2: — Diabetic foot Ulcer healing assessment scal. [file 40200_2015_167_MOESM2_ESM.docx]

| **First name:** .............................. **Last name:** ....................................... **Code:** ...................... | | | | | | | |
| --- | --- | --- | --- | --- | --- | --- | --- |
| 1 | **Method** | Routine dressing Olive oil dressing | | | | | |
| 2 | **Site of ulcer** | Sole Heel Dorsum of the feet Toe | | | | | |
| 3 | **Grade of ulcer** | Wagner 1 Wagner 2 | | | | | |
| 4 | **Size of ulcer** | Ulcer surface area: ................... cm^2^ Ulcer depth: ......................... cm | | | | | |
| 5 | **Duration of ulcer** | .................... months | | | | | |
| 6 | **Vascular status of ulcer** | Normal Abnormal | | | | | |
| 7 | **Neuropathy status of ulcer** | Normal Abnormal | | | | | |
| 8 | **Ulcer healing assessment scale** | **Week** | **Degree** | **Color** | **Surrounding tissues** | **Drainages** | **Total ulcer status** |
|  |  | **Baseline** |  |  |  |  |  |
|  |  | **After 1 week** |  |  |  |  |  |
|  |  | **After 2 weeks** |  |  |  |  |  |
|  |  | **After 3 weeks** |  |  |  |  |  |
|  |  | **After 4 weeks** |  |  |  |  |  |
| 9 | **Status of ulcer healing at the end of the study** | Complete Partial Lack of healing Deterioration | | | | | |
| 10 | **Adverse effects at the end of the study** | Ulcer area sensitivity Bleeding Infection Pain  Others: ........................................................................................................... | | | | | |

***Diabetic foot ulcer healing checklist***

***Diabetic foot Ulcer healing assessment scale***

| **Total score** | **Ulcer Parameters** | **Distribution of scores** | | | | | | | | | | | | | | | | |
| --- | --- | --- | --- | --- | --- | --- | --- | --- | --- | --- | --- | --- | --- | --- | --- | --- | --- | --- |
| **100** | **Degree** | **Stage** | **0** | **1** | **2** | **3** | **4** | **5** | **6** | **7** | **8** | **9** | **10** | **11** | **12** | **13** | **14** | **15** |
|  |  | **Score** | **100** | **90** | **90** | **80** | **75** | **65** | **65** | **55** | **50** | **40** | **40** | **30** | **25** | **15** | **15** | **10** |
| **100** | **Color** | **Center** | **Total healing** | | | **Red** | | | **Yellow** | | | **Necrotic** | | | **Necrotic + Red** | | | |
|  |  |  | **50** | | | **40** | | | **30** | | | **20** | | | **10** | | | |
|  |  | **Surroundings** | **Total healing** | | | **Red** | | | **Yellow** | | | **Necrotic** | | | **Necrotic + Red** | | | |
|  |  |  | **50** | | | **40** | | | **30** | | | **20** | | | **10** | | | |
| **100** | **Surrounding tissues** | **Color** | **Normal** | | | **Red** | | | **Pale** | | | **Cyanotic** | | | **-** | | | |
|  |  |  | **25** | | | **20** | | | **15** | | | **10** | | | **-** | | | |
|  |  | **Hotness** | **Yes** | | | **No** | | | **-** | | | **-** | | | **-** | | | |
|  |  |  | **0** | | | **25** | | | **-** | | | **-** | | | **-** | | | |
|  |  | **Edema** | **Yes** | | | **No** | | | **-** | | | **-** | | | **-** | | | |
|  |  |  | **0** | | | **25** | | | **-** | | | **-** | | | **-** | | | |
|  |  | **Sense** | **No** | | | **Decreased** | | | **Yes** | | | **-** | | | **-** | | | |
|  |  |  | **0** | | | **15** | | | **25** | | | **-** | | | **-** | | | |
| **100** | **Drainages** | **Color** | **Without Drainages** | | | **Serosal** | | | **Bloody** | | | **Yellow** | | | **Green** | | | |
|  |  |  | **40** | | | **30** | | | **30** | | | **20** | | | **10** | | | |
|  |  | **Odor** | **No** | | | **Yes** | | | **-** | | | **-** | | | **-** | | | |
|  |  |  | **20** | | | **0** | | | **-** | | | **-** | | | **-** | | | |
|  |  | **Amount** | **Without Drainages** | | | **Low** | | | **Moderate** | | | **Much** | | | **-** | | | |
|  |  |  | **40** | | | **20** | | | **20** | | | **10** | | | **-** | | | |
| **Total score (total healing) = 400 (more score= more healing)** | | | | | | | | | | | | | | | | | | |
